# Combined Aerobic and Resistance Training Increases Stretch- Shortening Cycle Potentiation and Walking Economy in Postmenopausal Women

**DOI:** 10.3389/fphys.2019.01472

**Published:** 2019-11-28

**Authors:** Harshvardhan Singh, Stephen J. Carter, Shannon L. Mathis, David R. Bryan, David M. Koceja, John P. McCarthy, Gary R. Hunter

**Affiliations:** ^1^Department of Physical Therapy, The University of Alabama at Birmingham, Birmingham, AL, United States; ^2^Department of Kinesiology, Indiana University Bloomington, Bloomington, IN, United States; ^3^Department of Kinesiology, The University of Alabama in Huntsville, Huntsville, AL, United States; ^4^Department of Nutrition Sciences, The University of Alabama at Birmingham, Birmingham, AL, United States

**Keywords:** exercise, walking, elastic energy, muscle contraction, potentiation, postmenopausal

## Abstract

**Purpose:**

Secondary analyses were performed to test whether combined aerobic and resistance training altered walking economy (i.e., net oxygen uptake) and/or stretch-shortening cycle potentiation (SSCP). A further objective was to determine if walking economy and SSCP were related before or after training.

**Methods:**

Ninety-two postmenopausal women were enrolled wherein 76 completed 16 weeks of supervised aerobic and resistance training. Participants were randomized to one of three training groups based on frequencies: (a) 1 d⋅wk^–1^ (*n* = 23); (b) 2 d⋅wk^–1^ (*n* = 30) or; (c) 3 d⋅wk^–1^ (*n* = 23). Following assessments were performed at baseline and post-training. Indirect calorimetry was used to measure maximal oxygen uptake (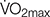) and walking economy (submaximal 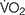 – resting 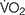 = net 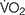) during a graded exercise test and steady-state treadmill task, respectively. SSCP was determined by measuring the difference between a concentric (CO) and counter-movement (CM) leg press throw.

**Results:**

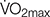
, walking economy, CO and CM velocity were significantly improved (*p* < 0.05) for all training groups, however; no time by group interactions were observed. Paired *t*-tests revealed participants exercise training 2 d⋅wk^–1^ exhibited a significant time effect for SSCP (+0.04 ± 0.09 ms^–1^; *p* = 0.03). At baseline, multiple linear regression showed a negative relationship between walking net 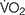 and SSCP (*r* = −0.22; *p* < 0.04) adjusted for relative proportion of  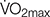. No such relationship was found post-training.

**Conclusion:**

Among older postmenopausal women, our results indicate that irrespective of frequency of training, 16 weeks of combined aerobic and resistance exercise training increased ease of walking and economy. Additionally, only participants exercising 2 d⋅wk^–1^ exhibited significant improvement in SSCP.

## Introduction

Obesity remains a major public health concern in the United States (World Health Organization, 2016). Given that habitual free-living physical activity (PA) is associated with favorable weight management (Schoeller et al., 1997; Weinsier et al., 2002), it is of interest to identify factors that may be influential to long-term PA adherence. Previously, we have shown that improved ease of walking (i.e., ↓heart rate for a given workload) increases spontaneous engagement in PA among older adults (Hunter et al., 2004a, b, 2005; Hartman et al., 2007), whereas improved walking economy, inverse of net walking 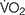 facilitates overall locomotion (Larew et al., 2003; Hunter et al., 2004a, b). Indeed, modifiable lifestyle factors like exercise training represent one of the most effective strategies to combat the loss of physiologic function with advancing age.

Resistance training, in particular, has been shown to improve ease of walking and economy, both of which, appear to enhance activity energy expenditure independent of exercise training (Hunter et al., 2000, 2001, 2013). As such, it is reasonable that walking/running economy may not only be a construct in athletic performance, but also a significant factor linked to non-exercise training activity thermogenesis (NEAT) - a key feature known to mitigate weight gain in older adults (Hunter et al., 2001, 2004b, 2013; Weinsier et al., 2002; Hunter and Byrne, 2005). Thus, a clearer understanding of factors that govern walking economy could be used to inform efficient exercise strategies to facilitate greater PA adherence among individuals that are overweight/obese.

Stretch-shortening cycle potentiation (SSCP) is one such factor that enhances locomotion economy, via increased utilization of elastic energy that leads to greater force-generation (Roberts, 2002). Prior research investigating SSCP has shown the muscle-tendon complex of the ankle joint is stretched during single support; then rapidly recoils during the push-off phase (Kubo et al., 1999, 2000; Fukunaga et al., 2001, 2002). Thus, the muscle-tendon complex of the ankle joint acts as a spring-like mechanism through the storage and release of elastic-strain energy during locomotion. It is known (Kurokawa et al., 2001) that longer achilles tendons have higher compliance in the muscle-tendon complex and greater ability to store and utilize mechanical energy. Thus, the spring-like effect of the muscle-tendon complex may reduce the energy needed for muscle shortening during locomotion. We have also reported that Achilles tendon length is positively associated with improved walking economy (McCarthy et al., 2006) - potentially through enhanced SSCP. Consistent with this premise, we have previously shown reduced net oxygen uptake (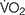) during running is positively associated with SSCP at the knee (Hunter et al., 2015). However, little is known about the relationship between SSCP at the knee and walking economy. This is specifically important for older populations, where there is an increased reliance on proximal muscles versus distal muscles for mobility (Silder et al., 2008).

Increased muscle strength is related to the generation of larger eccentric force of a stretch shortening cycle (McCarthy et al., 2012). Previously, we have shown that locomotion velocity is positively associated with large forces exerted during the late eccentric phase of a stretch shortening cycle (McCarthy et al., 2012). It is therefore plausible that at least one reason why resistance training increases walking and running economy is due to enhanced SSCP. This possibly occurs in young adults since a youthful muscle-tendon complex may possess greater elasticity, and thus, more potential for generating force through SSCP. Although adults lose elasticity with advancing age (Hsiao et al., 2015; Niyomchan et al., 2019), there is evidence that older adults may use SSCP to the same degree as young adults for the plantar flexor muscles (Svantesson and Grimby, 1995). This may, in part, explain the findings of improved walking economy following resistance training in older adults (Parker et al., 1996; Hartman et al., 2007; Fisher et al., 2013). However, the effect of resistance training on SSCP in older adults is currently unknown.

Therefore, the purpose of this study was to determine whether combined aerobic and resistance exercise training increases SSCP in postmenopausal women over the age of 60 years. Since little is known concerning the relationship between SSCP and walking economy, a secondary objective was to examine this relationship. We hypothesized that SSCP will increase following 16 weeks of combined aerobic and resistance training, whereas SSCP will be positively related to walking economy.

## Materials and Methods

### Participants

The present work is a secondary analysis of a study designed to delineate the effects of combined aerobic and resistance training following three different exercise frequencies over 16 weeks (Hunter et al., 2013). Initially, 92 postmenopausal women between 60 and 74 years of age provided baseline measures, wherein 76 participants completed the program. All participants were non-smokers and exercised less than one time per week, which was self-reported obtained during screening. Written informed consent was acquired from each participant prior to study involvement. All procedures confirmed to the guidelines set forth by the local (University of Alabama at Birmingham) institutional review board. Participants were randomly assigned to one of three training protocols: (a) Group 1: 1 d⋅wk^–1^ of resistance training and 1 d⋅wk^–1^ of aerobic training (1 + 1, *n* = 23); (b) Group 2: 2 d⋅wk^–1^ of resistance training and 2 d⋅wk^–1^ of aerobic training (2 + 2,*n* = 30) or; (c) Group 3: 3 d⋅wk^–1^ of resistance training and 3 d⋅wk^–1^ of aerobic training (3 + 3, *n* = 23).

### Body Composition

In accordance with customary procedures and manufacturer specifications, total body fat percent was estimated before and after the training program using dual-energy x-ray absorptiometry (Lunar DPX-L densitometer; LUNAR Radiation, Madison, WI, United States). Adult Software v1.33 was used to analyze scans.

### Resting Oxygen Uptake

Following an overnight fast between 1530 pm and 0800 am, resting 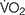 was measured via open circuit, indirect calorimetry system (DeltaTrac II: Sensor Medics, Yorba, CA, United States). The concluding 20 min of data were used for subsequent analyses.

### Maximal Aerobic Capacity and Walking Economy

A modified Balke treadmill test coupled with a metabolic cart (Max-1 Cart; Physio-Dyne Instrument Corporation, Quogue, NY, United States) was used to measure maximal aerobic capacity (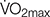). A 12-lead electrocardiogram and blood pressure measures (Omron Blood Pressure Monitor, model HEM-780; Omron Healthcare, Inc., Bannockburn, IL, United States) were taken at 2 min intervals during the test and recovery period. Under physician supervision, the treadmill test began with 2 min of walking at 0.89 m/s (2 mph). Grade was increased by +3.5% every 2 min until min 12 wherein grade was decreased to 12% and speed was increased to 1.34 m/s (3 mph). Later grade was increased again by +2.5% each min until exhaustion. Maximal heart rate, respiratory exchange ratio (RER), and 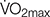 (mL⋅kg^–1^⋅min^–1^) were recorded as the highest 20 s averaged value.

On a separate day, walking economy was measured during a submaximal treadmill test (i.e., walking at 0.89 m/s). A walking speed of 0.89 m/s allowed us to achieve steady state within 4 min and compare walking economy at identical workloads. The 60 s mean value from the 3rd and 4th min were averaged to determine 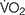. If 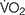 and/or heart rate increased during the 4 min – a 5th min was used. Net 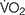 was calculated by subtracting resting 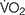 from steady-state 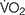.

### Maximum Muscle Strength Assessment

Participants performed a one-repetition maximum (1RM) test after the initial two exercise sessions (to permit overall familiarization). Notably we have previously revealed a high test-retest reliability for measurements conducted in our lab involving strength assessments (Hunter and Treuth, 1995). Determination of strength included the leg press, squats, leg extension, leg curl, elbow flexion, lateral pull-down, bench press, and military press. Lower back extension and bent leg sit-ups were performed with no weight according to section “Materials and Methods” previously described (Hunter and Treuth, 1995).

### Stretch-Shortening Cycle Potentiation

A ballistic leg press, corresponding with 100% total body weight used as resistance, respective for each participant (McCarthy et al., 2012), was performed pre- and post-training. Concentric (CO) velocity during a *static* leg press throw was performed by instructing participants to slowly lower the sled to 90° knee flexion and hold the position for 3 s before pushing (i.e., extending the knees) the sled as fast as possible for maximal force. Concentric velocity was also measured during a counter-movement (CM) leg press throw by instructing participants to lower the sled to 90° knee flexion then immediately push sled off feet as fast as possible. The leg press sled was connected to a linear position transducer which was synced with a National Instruments system with a customized LabVIEW (Laboratory Virtual Instrument Engineering Workbench, version 7.1) software connected to a 16 channel 12-bit data acquisition system. Before each testing, calibration of linear position transducer was performed. Data was collected at 1 kHz. A low-pass fourth order Butterworth filter was applied with a cutoff frequency of 50 Hz. The linear position transducer tracks the linear position of the leg press sled. Using finite-difference technique the displacement data of sled was used to calculate velocity (McCarthy et al., 2012). The difference between CO and CM was representative of SSCP.

### Supervised Exercise Training

Participants completed a 3–4 min warm-up on a treadmill or cycle ergometer followed by 3–4 min of stretching prior to each exercise session. All training sessions were supervised by an exercise physiologist in a facility dedicated to research. Each session lasted ≈50 min. The mode of aerobic exercise included a treadmill or cycle ergometer with at least 50% of training completed on the treadmill. Week one commenced with 20 min of continuous aerobic exercise corresponding with ≈67% of maximal heart rate (MHR). Weekly volume of aerobic exercise volume was progressively increased by modifying the duration first followed by intensity. For instance, duration was increased in weeks 2–5 by 5 min each week to reach a total of 40 min per session. At week 6, intensity was increased to ≈71% MHR, while duration decreased to 30 min. Duration then returned to 40 min by week 7. In week 8, intensity increased to ≈75% MHR while duration decreased to 30 min. At week 9, duration returned to 40 min. Intensity increased to ≈80% of MHR for 30 min by week 10. In week 11, duration increased to 40 min. For the remaining sessions, participants trained at ≈80% of MHR for 40 min. For subjects who did an aerobic and resistance workout same day, order of aerobic and resistance was alternated on their subsequent session. Rest interval between the sessions of aerobic and resistance training was 5–10 min and for resistance training it was 1–2 min between each set.

The resistance training protocol began with two sets of 10 repetitions at an intensity matching 60% 1RM with 90 to 120 s rest period between sets. Exercises included leg press, squats, leg extension, leg curl, elbow flexion, lateral pull-down, bench press, military press, lower back extension, and bent leg sit-ups. Resistance training progressed to 80% 1RM at week 8 (Hunter and Treuth, 1995).

### Statistical Analyses

Baseline descriptive measures are reported as average ± SD. Effects on CO velocity, CM velocity, 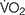 max and net 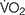 from the three exercising training frequencies incorporating combined aerobic and resistance training were determined via two-way (group by time) repeated-measures analysis of variance (ANOVA). A One-way ANOVAs was used to compare age, height, body mass, body fat%, and heart rate. Multiple linear regression analysis (enter method) was used to determine associations of walking economy with groups, SSCP, and relative exercise intensity (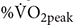) during the walking task at baseline and post-training. Because of equipment malfunction, there is missing data for variables such as CM velocity, CO velocity, 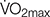, and net 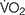. Paired *t*-tests were run to analyze differences in SSCP for each respective groups. Degrees of freedom were calculated to reflect missing data. The threshold of statistical significance was set *a priori* with the *p*-value ≤ 0.05 for all analyses.

## Results

**FIGURE 1:**
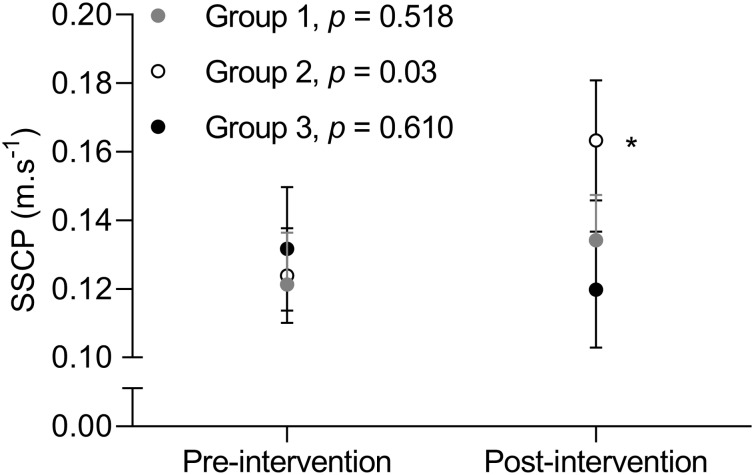
Mean ± standard error of group values for stretch-shortening cycle potentiation (SSCP) captured by the pre-training versus post-training difference in a concentric only and counter-movement leg press throw. Note the significant increase in group 2 where: Group 1: 1 d⋅wk^–1^ of resistance training and 1 d⋅wk^–1^ of aerobic training (*n* = 23); Group 2: 2 d⋅wk^–1^ of resistance training and 2 d⋅wk^–1^ of aerobic training (*n* = 30); Group 3: 3 d⋅wk^–1^ of resistance training and 3 d⋅wk^–1^ of aerobic training (*n* = 23). ^∗^*p* = 0.03.

Baseline descriptive measures are shown in Table 1. Pre-/post-training data, including mean and SD’s, for all study variables are presented in Table 2. There were no differences in age [*F*(2,73) = 1.8] or height [*F*(2,73) = 1.2] across the three exercise training groups. Body mass was significantly decreased [Time *F*(1,71) = 4.24, Group *F*(2,71) = 4.51, Time × Group *F*(2,71) = 3.49] post-training. Body fat percent was significantly decreased [Time *F*(1,71) = 32.0, Group *F*(2,71) = 4.0, Time × Group *F*(2,71) = 2.7] post-training, however no time by group interactions were observed. Maximal aerobic capacity, as evidenced by 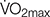 was significantly increased [Time *F*(1,65) = 8.6, Group *F*(2,65) = 3.7, Time × Group *F*(2,65) = 1.4) among all groups. Likewise, walking economy (i.e., net 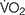) [Time *F*(1,69) = 12.5, Group *F*(2,69) = 0.12, Time × Group *F* (2,69) = 0.95] and heart rate [Time *F*(1,69) = 63.6, Group *F*(2,69) = 1.5, Time × Group *F*(2,69) = 0.95] decreased similarly between groups across time. Despite no apparent time by group interactions for CO velocity [Time *F*(1,65) = 6.5, Group *F*(2,65) = 0.08, Time × group *F*(2,65) = 2.87] and CM velocity [Time *F*(1,65) = 19.0, Group *F*(2,65) = 0.3, Time × Group *F*(2,65) = 0.2, *df* = 65], a significant time effect (*p* < 0.01 for both) was revealed for all the groups. Paired *t*-tests analyses indicated a significant increase in SSCP in Group 2 only (Figure 1; Group 2, *p* = 0.03; Groups 1 and 3, *p* > 0.5).

**TABLE 1:** Participant characteristics at baseline (*n* = 92).

** Characteristic**	**Mean ± SD**
Age (year)	65 ± 4
Height (m)	1.65 ± 0.06
Body mass (kg)	73.8 ± 11.4
Body fat (%)	42.6 ± 6.1
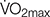 (mL⋅kg^–1^⋅min^–1^)	22.8 ± 4.6
CM velocity (ms^–1^)	0.90 ± 0.12
CO velocity (ms^–1^)	0.77 ± 0.12
net 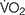 (mL⋅kg^–1^⋅min^–1^)	7.3 ± 1.28

*CM, countermovement; CO, concentric only; 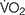, oxygen uptake.*

**TABLE 2:** Differences in physiological and functional parameters for each respective groups.

	**Group 1^a^**			**Group 2^b^**			**Group 3^c^**			
**Parameters**	***n* = 23**			***n* = 30**			***n* = 23**			
				
	**Pre**	**Post**	***% diff***	**Pre**	**Post**	***% diff***	**Pre**	**Post**	***% diff***	***p*-value**
Age (yr)	66 ± 4			64 ± 3			64 ± 3 (*n* = 21)			*G* = 0.17
Height (cm)	167 ± 6			165 ± 5			164 ± 4 (*n* = 21)			*G* = 0.31
Body mass (kg)	76.3 ± 11.4	76.4 ± 11.4	0.13	74.9 ± 9.4	73.7 ± 9.1	1.6	67.8 ± 9.6 (*n* = 21)	67.60 ± 9.7	0.29	*T* < 0.05^∗^ *G* < 0.02^∗^ G×T < 0.04
Body fat (%)	44.1 ± 5.3	43.6 ± 5.7	1.13	42.8 ± 4.7	41.1 ± 4.8	3.97	40.1 ± 5.8 (*n* = 21)	38.5 ± 6.7	3.99	*T* < 0.01^∗^ *G* = 0.02^∗^ G×T = 0.07
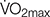 (mL⋅kg^–1^⋅min^–1^)	21.0 ± 2.9	21.7 ± 3.7	3.33	23.0 ± 4.6 (*n* = 26)	24.9 ± 3.8	8.2	24.0 ± 4.3 (*n* = 19)	24.5 ± 5.1	2.08	*T* < 0.01^∗^ *G* = 0.03 G×T = 0.25
CM velocity (m.s^–1^)	0.89 ± 0.12 (*n* = 22)	0.93 ± 0.12	4.49	0.91 ± 0.10 (*n* = 25)	0.96 ± 0.10	5.5	0.88 ± 0.11 (*n* = 21)	0.93 ± 0.13	5.68	*T* < 0.01^∗^ *G* = 0.73 G×T = 0.82
CO velocity (m.s^–1^)	0.78 ± 0.13 (*n* = 22)	0.80 ± 0.12	2.56	0.78 ± 0.10 (*n* = 25)	0.78 ± 0.13	0.0	0.75 ± 0.14 (*n* = 21)	0.81 ± 0.11	8.0	*T* < 0.01^∗^ *G* = 0.61 G×T = 0.21
net 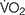 (mL⋅kg^–1^⋅min^–1^)	7.5 ± 1.5	6.6 ± 0.9	12.0	7.3 ± 1.3 (*n* = 29)	6.7 ± 1.5	8.22	7.1 ± 1.1 (*n* = 20)	6.7 ± 1.5	5.63	*T* < 0.01^∗^ *G* = 0.89 G×T = 0.64
Heart rate (beats⋅min^–1^)	108 ± 14	97 ± 8	10.18	101 ± 14 (*n* = 26)	94 ± 14	6.93	101 ± 12	94 ± 10	6.93	*T* < 0.01^∗^ *G* = 0.23 G×T = 0.39

*CM, countermovement; CO, concentric only; 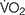, oxygen uptake. Data are presented as means ± standard deviations. ^∗^*p* < 0.05. Group differences (G); Time differences (T); Group × Time Interaction (G × T). ^*a*^1 d⋅wk^–1^ of resistance training and 1 d⋅wk^–1^ of aerobic training; ^*b*^2 d⋅wk^–1^ of resistance training and 2 d⋅wk^–1^ of aerobic training, and ^*c*^3 d⋅wk^–1^ of resistance training and 3 d⋅wk^–1^ of aerobic training. *%diff*, percent difference.*

Multiple linear regression of baseline measures (*n* = 92; Table 3) showed SSCP and relative exercise intensity (%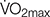) were independently related to net 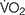 while walking at 2 mph (*r*_partial_ = −0.22, *p* < 0.04; and *r*_partial_ = 0.54, *p* < 0.01; respectively). However, only %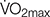 was significantly related to net 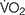 post-training after adjusting for different exercise training groups (*r*_partial_ = 0.49, *p* < 0.01). No relationship was observed between SSCP and %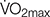 post-training (*r*_partial_ = 0.18, *p* = 0.12).

**TABLE 3:** Multiple linear regression model of walking net 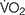 versus stretch-shortening cycle potentiation (SSCP) and relative exercise intensity (%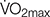) at baseline (*n* = 92) and post-training (*n* = 68).

**Independent variable**	**Dependent variable**	***R*^2^**	**Intercept**	**Slope**	***r*_partial_**	***p-value***
*Model 1:* Baseline net 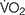 walk (mL⋅kg^–1^⋅min^–1^)		0.32	5.0			<0.01
	SSCP			–2.88	–0.22	<0.04
	Percentage 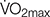			6.00	0.54	<0.01
*Model 2:* Post-training net 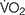 walk (mL⋅kg^–1^⋅min^–1^)		0.25	3.2			<0.01
	SSCP			2.37	0.18	0.12
	Percentage 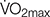			7.84	0.49	<0.01

*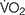, oxygen uptake; SSCP, stretch-shortening cycle potentiation. No relationship with groups were found.*

## Discussion

The primary objective was to determine whether combined aerobic and resistance exercise training increases SSCP in postmenopausal women over the age of 60 years. A secondary point of interest was to determine whether SSCP was related to walking economy (i.e., net 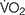) at 0.89 m/s. Results revealed, independent of weekly volume, that 16 weeks of combined exercise training significantly improved both walking economy and ease (i.e., ↓heart rate). Although participants exercising 1 d⋅wk^–1^ and 3 d⋅wk^–1^ did not increase SSCP, significant improvement was detected among participants exercising 2 d⋅wk^–1^. Of note, SSCP was related to walking economy at baseline suggesting that the ability to use elastic energy, as evidenced by SSCP, may partially lower the energetic requirements for non-graded walking. These findings are consistent with previous work in our lab wherein SSCP related to non-graded walking (unpublished data) and running economy in young (≈31 year) men (Hunter et al., 2015). To our knowledge, we are the first to show the relationship of SSCP to walking economy in an older population. However, we were surprised to discover this relationship did not persist following exercise training, thus we offer several possible explanations for this observation.

Whereas post-training walking economy was increased among all groups, no relationship between SSCP and walking economy was noted – yet this is not entirely unexpected. A possible explanation involves the muscle groups/patterns used during non-graded walking in older individuals. Stretch-shortening cycle potentiation was measured incorporating a leg press throw, a task primarily executed from the hip and knee extensors. Hence, in this study, it is probable that the technique used to measure SSCP was primarily developed in the hip/knee extensors (as opposed to the plantar flexors). Evidence suggests older adults tend to be more dependent on hip extension and less on plantar flexion during walking tasks compared to younger adults (Silder et al., 2008), a feature likely due to reduced strength in the plantar flexor muscles (Silder et al., 2008; Anderson and Madigan, 2014). Given that hip/knee extensors bioenergetics are less efficient than plantar flexors, greater dependence on hip/knee extension may contribute to an increase in energy expenditure (Seki et al., 2019). Though speculative, the improved muscle strength post-training may have enabled the older adults to adopt a walking pattern that more closely resembles that of younger adults (i.e., shift in the involvement of distal muscles and respective reliance on elastic energy from distal muscle-tendon units).

There is evidence that indicates older adults have insufficient range of motion at the hips which could contribute to the known age-related deficits in gait (Anderson and Madigan, 2014). It is possible that the combined aerobic and resistance training could have positively affected hip/knee range of motion (ROM), thus enhancing walking economy, but this is only speculative as no direct measures were taken of ROM. Based on these factors, it is reasonable that the potential link between SSCP (reflective of knee/hip extensors) and walking economy would be expected to diminish post-training. Moreover, effects of exercise training are dictated by multiple factors which can affect within-individual response to different types of exercise training (Hecksteden et al., 2015). Further examination of factors including altered biomechanics and change in achilles tendon compliance (Kooper et al., 2014) in various study designs such as crossover design should be considered to further refine our understanding linking SSCP and walking economy.

Indeed, both ease of walking and economy were improved following 16 weeks of combined aerobic and resistance training in the present study. Since all the participants performed a combination of both aerobic *and* resistance training, we are unable to differentiate which mode of exercise training is better suited to enhance ease of walking and economy. This is in line with prior evidence showing positive effects on walking economy due to multicomponent exercise training (Valenti et al., 2016). Both these modes of exercise, aerobic and resistance training, can lead to improved mitochondrial profile and function (Nilsson and Tarnopolsky, 2019), which may improve walking economy. However, there is proof that positive relationship exists between stage 3 respiration (coupled respiration) and stage 4 respiration (uncoupled respiration) with both, pre and post aerobic training, suggesting that mitochondrial function has little effect on walking economy due to aerobic training (Hunter et al., 2005, 2019). These findings may suggest that factors other than mitochondrial efficiency factors influence improvements in walking economy following exercise training.

Co-contraction of antagonist muscles is inversely related with walking economy in older adults (Ortega and Farley, 2015). Exercise training can reduce antagonist muscle activation in older adults (Hakkinen et al., 1998), however it is undecided if reduced co-contraction of antagonist muscles can improve walking economy in older adults (Beck et al., 2018). We did not assess co-contraction of muscle in our current study. Notably, several studies have shown increased ease of walking and economy with resistance training – independent of improved aerobic capacity (Parker et al., 1996; Hartman et al., 2007). Further evidence involving changes in muscle strength following resistance training have been associated with increased walking economy (Hunter et al., 2008). Thus, our findings underline the importance of combined aerobic and exercise training to elicit meaningful improvements in ease of walking and economy - particularly among older adults. This information has important clinical application/utility as ease of walking and economy are associated with both increased free-living energy expenditure (Hunter et al., 2000, 2013) and longer-term accretion of body fat (Weinsier et al., 2002). Certainly these data offer a framework for further investigation to incorporate exercise training as a means to augment ease of walking and economy. This, in turn, may enhance spontaneous engagement in PA among older adults.

We were not entirely surprised with our finding of increased SSCP only with group 2. A recent meta-analysis suggested that two exercise training sessions consisting of resistive exercises per week is most efficient for improving muscle strength in older adults (Borde et al., 2015). Since muscle strength can, in part, dictate improvements in SSCP, it may explain increased SSCP in Group 2 only. Further, effects of exercise could be sex-dependent (Hunter and Treuth, 1995). Older women (who were the study population of our study) may respond better to moderate exercise frequency (Grgic et al., 2018). Notably, we used a combination of aerobic and resistance training. There is lack of data on effects of different types of exercise training, specifically a combination of aerobic and resistance training, on SSCP. Future studies should examine effects of different types of training with different frequency/volume on SSCP in older women.

## Conclusion

Among older postmenopausal women, our results indicate that irrespective of frequency of training, 16 weeks of combined aerobic and resistance exercise training increased ease of walking and economy. Additionally, only participants exercising 2 d⋅wk^–1^ exhibited significant improvement in SSCP. Baseline measures of SSCP were associated with walking economy independent of relative exercise intensity, however, these results did not persist post-training. Individual responsiveness to exercise training and shifts in muscle involvement/patterns may have contributed to these findings.

## Data Availability Statement

The datasets related to this study are available on request to the corresponding author.

## Ethics Statement

The studies involving human participants were reviewed and approved by the Institutional Review Board, University of Alabama at Birmingham. The patients/participants provided their written informed consent to participate in this study.

## Author Contributions

GH, JM, and DB were responsible for study design and data acquisition. All authors helped with data analysis and data interpretation, critically revised the manuscript, contributed with important intellectual content, approved the final version of the article to be submitted for publication purpose, and took responsibility for appropriate portions of the content. HS, GH, and SM wrote the first draft of the manuscript.

## Conflict of Interest

The authors declare that the research was conducted in the absence of any commercial or financial relationships that could be construed as a potential conflict of interest.
